# Dr. Theodore Dimon

**Published:** 1889-09

**Authors:** 


Dr. Theodore Dimon, of Auburn, N. Y., died July 22, 1889, aged seventy-two years. He was formerly superintendent of the Asylum for Insane Criminals at Auburn, and medical officer of the State prison. He was a regimental surgeon in the late war, and a member of the Military Order of the Loyal Legion at the time of his death. Dr. Dimon was an influential member of the profession, an active worker in medical societies, and a forcible writer.

